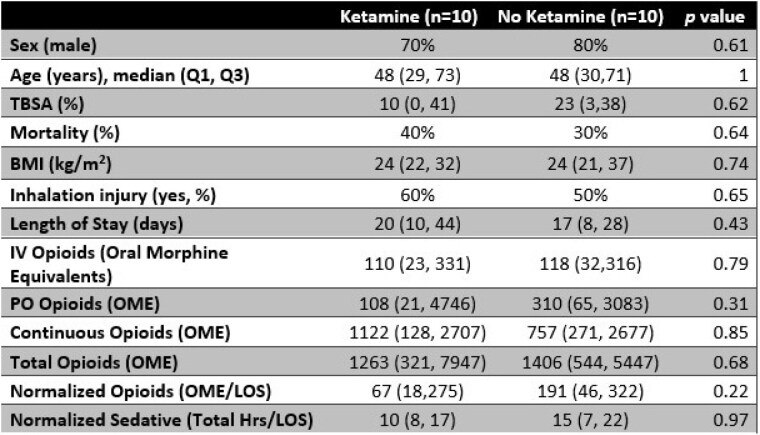# 575 The Effect of Ketamine Infusions on Sedation and Opioid Analgesia in Critically Ill Burn Patients

**DOI:** 10.1093/jbcr/iraf019.204

**Published:** 2025-04-01

**Authors:** Evan Barrios, Maria Herrera-Rodriguez, Clay Rodriguez, Andrea Munden, Amalia Cochran, Ian Driscoll

**Affiliations:** University of Florida; Florida Atlantic University; University of Florida; University of Florida; University of Florida; University of Florida College of Medicine

## Abstract

**Introduction:**

Ketamine is commonly used as a continuous infusion to provide sedation and analgesia in critically ill patients. Data comparing sedative and opioid usage in ketamine and non-ketamine patient cohorts is limited in the burn literature. We hypothesized that patients receiving continuous ketamine infusions (CKI) also receive less opioids over the course of their hospitalization.

**Methods:**

This is a single-center retrospective review of adult patients admitted to an ABA-verified Burn center. The first cohort (n=10) spans June 2022—May 2023 in which ketamine use was restricted due to shortage, and the second cohort (n=10) is from August 2020—March 2022 when ketamine infusions were routinely used. Only patients who received continuous ketamine infusions were considered for this cohort.

**Results:**

CKI did not result in decreased administration of intermittent parenteral opioid (110 Oral Morphine Equivalents [OME] vs 118 OME, p=0.79), oral opioid (108 OME vs 310 OME, p=0.31), continuous parenteral opioid infusions (1122 OME vs 757 OME, p=0.85), or total opioid (1265 OME vs 1406 OME, p=0.68) in patient cohorts which appeared otherwise similar (Table 1). Interestingly, the number of hours that patients received continuous parenteral sedative infusions (normalized for length of stay) may be increased in the CKI cohort (19.4 hours of sedative per day of hospitalization vs 15.3, p=0.09) although this was not statistically significant.

**Conclusions:**

Larger patient cohorts are required to appropriately power studies to detect differences in analgesic and sedative agents in critically injured burn patients. Ketamine continues to function as a crucial adjunct when considering the difficult prospect of pain control and appropriate sedation. Failure to decrease opioid administration in the patients receiving ketamine may reflect the difficulty assessing pain control in these critically ill patients.

**Applicability of Research to Practice:**

Ketamine is commonly used in the Burn ICU. Its effects on agents with risk dependency (such as opioids) or those with potential side effects (such as antipsychotic drugs) are currently understudied.

**Funding for the Study:**

N/A